# Isolation, Mutagenesis, and Organic Acid Secretion of a Highly Efficient Phosphate-Solubilizing Fungus

**DOI:** 10.3389/fmicb.2022.793122

**Published:** 2022-04-25

**Authors:** Tianyou Yang, Linbo Li, Baoshi Wang, Jing Tian, Fanghao Shi, Shishuang Zhang, Zhongqi Wu

**Affiliations:** ^1^School of Life Science and Technology, Henan Institute of Science and Technology, Xinxiang, China; ^2^Sino-Danish Center, University of Chinese Academy of Sciences, Beijing, China

**Keywords:** phosphate-solubilizing fungi, low-energy ion implantation mutagenesis, mutants, organic acids, sustainable agriculture

## Abstract

The highly effective phosphate-solubilizing microorganisms are significant for making full use of the potential phosphorus resources in the soil and alleviating the shortage of phosphorus resources. In this study, a phosphate-solubilizing fungus was isolated from wheat and cotton rhizosphere soils in the lower reaches of the Yellow River in China and was identified as *Penicillium oxalicum* by morphological and ITS sequencing analysis. In order to obtain a fungus with more efficient phosphorus solubilization ability, we tested three positive mutant strains (P1, P2, and P3) and three negative mutant strains (N1, N2, and N3) through low-energy nitrogen ion implantation mutagenesis. Compared with the parental strain, the phosphate-solubilizing capacity of P1, P2, and P3 was enhanced by 56.88%, 42.26%, and 32.15%, respectively, and that of N1, N2, and N3 was weakened by 47.53%, 35.27%, and 30.86%, respectively. Compared with the parental strain, the total amount of organic acids secreted significantly increased in the three positive mutant strains and decreased in the negative mutant strains; the pH of culture medium was significantly lower in the positive mutant strains and higher in the negative mutant strains. The capacity of phosphate-solubilizing fungus to secrete organic acids and reduce the growth-medium pH was closely related to its phosphate-solubilizing ability. The changes in the amount of organic acids secreted by mutants can alter their acidification and phosphate-solubilizing capacity. In conclusion, this study offers a theoretical basis and strain materials for the exploration and application of phosphate-solubilizing fungi.

## Introduction

Phosphorus is an essential nutrient element for crop growth and development (Yongbin et al., 2020). There is little available inorganic phosphorus to be absorbed and utilized by crops in the soil, whereas the majority of phosphorus is in insoluble forms that crops cannot utilize (Busato et al., 2017). Given low phosphorus availability but abundant potential phosphorus sources in the soil, finding and characterizing phosphate-solubilizing microorganisms to make full use of potential phosphorus resources in the soil has profound strategic significance for alleviating the shortage of phosphorus resources and developing sustainable and efficient agriculture (Sharma et al., 2013). At present, the research on phosphate-solubilizing microorganisms mostly focuses on isolation (Shi et al., 2014; Tomer et al., 2017), screening, and identification (Chakdar et al., 2018). There is a lack of highly efficient phosphate-solubilizing microorganisms, and the underlying phosphate-solubilizing mechanisms are poorly understood, thus restricting the utilization of phosphate-solubilizing microorganisms (Antoun, 2012). Using mutation to produce highly efficient phosphate-solubilizing microorganisms and characterization of phosphate-solubilizing mechanisms have always been important research topics in the field of soil microorganisms and biofertilizers (Gong et al., 2014). Both acid production and phosphate solubilization by phosphate-solubilizing microorganisms can be enhanced by radiation mutagenesis (Busato et al., 2017). Silva et al. (2014) induced mutation of *Aspergillus niger* by UV and found that the phosphate-solubilizing ability of mutant FS1-331 increased by up to 2.50 times compared with that of the original strain, with an increase in organic acid production being an important reason for improved phosphate-solubilizing ability. Moreover, Li et al. (2011b) studied the effect of microwave radiation on *Klebsiella pneumonia* RSN19 and showed that it could greatly enhance the nitrogen-fixing and phosphate-solubilizing capacities of the strain.

Since its application in biological mutation and genetic breeding in the 1980s, low-energy ion beam mutagenesis, characterized by a unique radiation effect, has been widely applied in microbial breeding (Li et al., 2013). In recent years, low-energy ion beam mutagenesis for phosphate-solubilizing microorganisms has been studied extensively. For example, You et al. (2009) mutated *Bacillus subtilis* P-1 using nitrogen ion beam implantation, and one phosphate-solubilizing mutant strain was obtained, with phosphate-solubilizing ability enhanced by 48%. Peng et al. (2020) adopted ion beam implantation technique in the breeding of phosphate-solubilizing mutant strain of *Bacillus subtilis* LA, resulting in the improved phosphate-solubilizing ability. Low-energy ion beam is a new type of mutation source (Semsang et al., 2018) with four mutagenic effects (energy exchange, energy deposition, mass deposition, and charge exchange), little induced damage, a wide spectrum of mutations, and a high mutation rate (Maekawa et al., 2003; Okamura et al., 2003; Yamaguchi et al., 2003; Li et al., 2007). Currently, the mutagenic materials in ion beam mutagenesis are mostly phosphate-solubilizing bacteria, and there are few studies on the ion beam mutagenesis in fungi (Nguyen and Bruns, 2015). Ion beam mutagenesis for phosphate-solubilizing fungi is expected to result in the formation of highly efficient phosphate-solubilizing fungal mutants (Semsang et al., 2018), which is of great significance for the breeding of phosphate-solubilizing strains and the characterization of the underlying phosphate-solubilizing mechanisms.

There are four main mechanisms involved in inorganic phosphate solubilization by microorganisms, including secretion of organic acids, hydrogen protons, proteins, and chelation (Khan et al., 2014). Gong et al. (2014) isolated one strain of *Penicillium* sp. PSM11-5 from an alum mine and its phosphate-solubilizing effect was closely related to the secretion of gluconic acid and citric acid. Ahuja et al. (2007) argued that the citric acid and oxalic acid produced by *Paecilomyces marquandii* AA1 were important in phosphate solubilization. Mendes et al. (2013) found that citric acid, gluconic acid, and oxalic acid secreted by *Penicillium canescens* were the main mechanisms for solubilizing calcium phosphate. In general, the organic acids found to be involved in phosphate solubilization include oxalic (Mendes et al., 2020), citric, lactic, acetic, oxaloacetic, etc. (Parsons and Fry, 2012; Della Monica et al., 2018).

In this study, phosphate-solubilizing fungi in the rhizospheric soil of crops were screened to obtain highly efficient phosphate-solubilizing mutants using ion beams, and the underlying phosphate-solubilizing mechanisms were explored from the perspective of secretion of organic acid, for example, lowering pH and chelating calcium ions to release phosphate. This study is aimed to provide biological materials and a theoretical basis for the development of highly efficient phosphate-solubilizing fungi and the characterization of phosphate-solubilizing mechanisms.

## Materials and Methods

### Isolation and Screening of Phosphate-Solubilizing Fungi

The rhizospheric soil samples of wheat and cotton were collected from the Henan Institute of Science and Technology experimental field (longitude: 113.77° latitude: 35.46°) located at downstream region of the Yellow River of China. Five grams of rhizospheric soil was mixed evenly in 50 ml of sterile water using a shaker and then left to stand. The supernatant was collected and serially diluted (10^−4^, 10^−5^_,_ and 10^−6^). 0.20 ml of each fungi dilution was spread onto the solid PSFM and cultured at 28°C for 5 days, with five replicates in each treatment. Then, fungal colonies with obvious phosphate-solubilizing (clear) zones around them were selected, transferred to PDA slants, and stored at 4°C. The isolated phosphate-solubilizing fungi were cultured in the PSFM for 120 h, and the diameter of colonies (*D*_colonies_) and the diameter of surrounding phosphate-solubilized zones (*D*_solvent zones_) were measured. The fungal colonies with *D*_solvent zones_/*D*_colonies_ > 1.1 were transferred to PDA slants and stored at 4°C. Then, spores from slants of phosphate-solubilizing fungi that had the above ratio > 2 were taken into 100 ml of PSFM and cultured on a shaker at 70 r·min^−1^ and 28°C for 5 days, with three olybdenum-stibium colorimetry method (Zhang et al., 2018), and the most efficient strains of phosphate-solubilizing fungi were selected as the test strains.

### Identification of Phosphate-Solubilizing Fungi

The test strains were inoculated onto the PDA medium and cultured at 28°C for 2 days, and the colony morphology was observed. Potato dextrose agar (PDA) medium was composed of 200 g of potato, 20 g of sucrose, 20 g of agar, and 1,000 ml of distilled water (pH 7.00). The spores of *P. oxalicum* F9 were picked with a sterile toothpick and inoculated onto a PDA plate. A 1 cm^2^ metal sheet was inserted near the inoculation point, followed by culturing at 28°C. After the mycelia climbed on the metal sheet, they were observed under a Quanta 200 environmental scanning electron microscope (FEI Company, United States).

In the ITS sequence analysis, the *P. oxalicum* F9 was cultured on the PDA medium, and the mycelia on the surface of the dish were scraped off and ground in a mortar with liquid nitrogen. The genomic DNA was extracted from the ground mycelia using a Fungal DNA Extraction Kit (Sangon Biotech, Shanghai). The universal primers for the fungal ITS are: forward: ITS1 5’-TCCGTAGGTGAACCTGCGG-3′ and reverse: ITS4 5’-TCCTCCGCTTATTGATATGC-3′. The universal primers for fungal internal transcript space (ITS) sequence amplification were synthesized by TaKaRa Bioengineering (Dalian) Co., Ltd. The PCR products were sent to TaKaRa Bioengineering (Dalian) Co., Ltd., for sequencing, and the DNA sequences were obtained. Finally, the sequences were compared and analyzed through BLAST in GenBank, and the phylogenetic tree of *P. oxalicum* F9 was constructed using MEGA7.0 software.

### Determination of pH of Phosphate-Solubilizing Fungal Culture Medium

PSFM liquid (1 ml) of phosphate-solubilizing fungi was taken and centrifuged at 8,000 r·min^−1^ for 5 min. The pH of supernatant was measured by pH meter. Phosphate-solubilizing fundamental medium (PSFM) was composed of 10 g of glucose, 0.30 g of NaCl, 0.30 g of KCl, 0.30 g of MgSO_4_·7H_2_O, 0.03 g of FeSO_4_·7H_2_O, 0.03 g of MnSO_4_·4H_2_O, 0.50 g of (NH_4_)_2_SO_4_, 5 g of Ca_3_(PO_4_)_2_, and 1,000 ml of distilled water (pH 7.00–7.50). Ca_3_(PO_4_)_2_ was added after being fully ground and sterilized. Solid PSFM was supplemented with 15 g·L^−1^ agar.

### Ion Beam Mutagenesis for Phosphate-Solubilizing Fungus *Penicillium oxalicum* F9

Evenly mixed *P. oxalicum* F9 spore suspension (0.20 ml, 10^8^ CFU·ml^−1^) was spread in a clean and sterile Petri dish, placed on a super clean bench and blown dry with sterile air to prepare the *P. oxalicum* F9 spore membrane. The Petri dish was placed in a microbial target chamber of a Titan ion implanter, followed by radiation mutagenesis with low-energy nitrogen ions (20, 25, and 30 keV) at a radiation dose of 5 × 10^14^, 1 × 10^15^, 5 × 10^15^, and 1 × 10^16^ ions·cm^−2^, including the vacuum controls. After implantation, the bacterial membrane was rinsed with 1 ml of sterile water in a test tube, thoroughly dispersed and mixed, diluted to 10^−3^, 10^−4^_,_ and 10^−5^, and then spread onto a PSFM plate. The survival rate of *P. oxalicum* F9 was calculated as:

*Penicillium oxalicum* F9 was screened primarily based on *D*_solvent zones_/*D*_colonies_ and rescreened *via* PSFM culturing on a shaker at 28°C for 5 days. The phosphate-solubilizing ability of fungus strains was detected *via* molybdenum-antimony anti-colorimetry.

### Determination of Organic Acids in Phosphate-Solubilizing Fungal Culture Medium

*Penicillium oxalicum* F9 culture medium (1 ml) was placed into a centrifuge tube and centrifuged at 8,000 r·min^−1^ for 5 min. The supernatant was filtered through a 0.22-μm filter membrane and injected by a 1 ml syringe into an ICS-2000 ion chromatograph (DIONEX, United States) for detection using a DIONEX IonPac®AS11-HC anion-exchange column (aqueous; sample flow rate: 1.20 ml·min^−1^, column temperature: 30°C, injection volume: 0.50 ml). The chromatographic data were analyzed according to the instructions using a Chromeleon chromatographic working station.

### Data Analysis

All tests and treatments were performed with three repetitions, and the values were expressed as the mean of them. Pearson correlation analysis was used to evaluate whether there was a significant relationship in phosphate-solubilizing ability of fungus and the culture medium pH. The significant data were compared using the LSD test of one-way ANOVA (*p* < 0.05).

## Results

### Screening of Phosphate-Solubilizing Fungus

**Figure 1:**
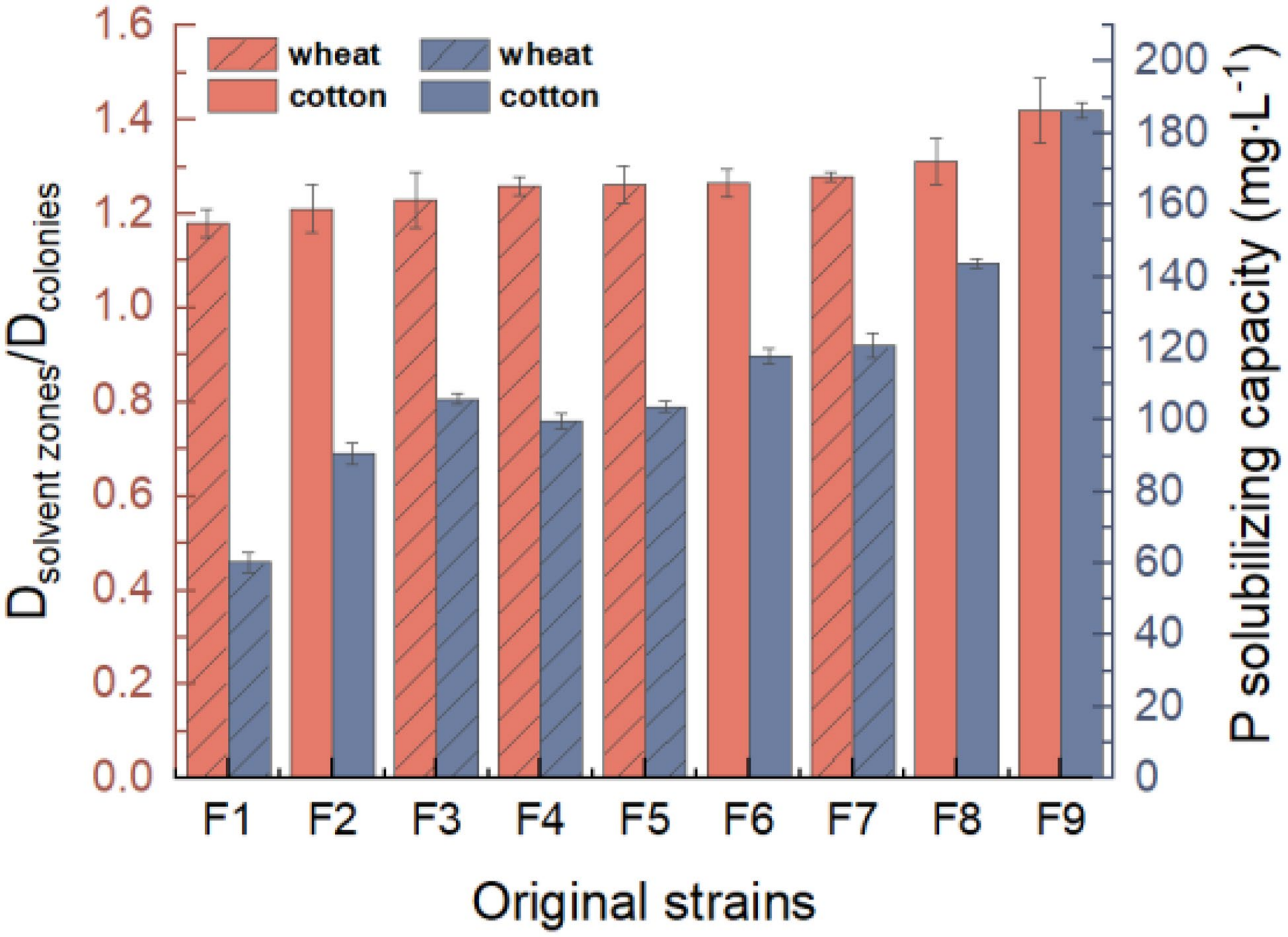
Screening of phosphate solubilizing fungi from rhizospheric of crops. The red represents *D*_solvent zones_/*D*_colonies_ value, and the blue represents P-solubilizing capacity.

Nine strains (F1, F2, F3, F4, F5, F6, F7, F8, and F9) of phosphate-solubilizing fungi were isolated *via* the primary screening and rescreening based on the *D*_solvent zones_/*D*_colonies_ (Figure 1). All of them could grow on the PSFM plate with tricalcium phosphate as the only phosphorus source and could produce obvious halo zones. The *D*_solvent zones_/*D*_colonies_ of F1 was the lowest (1.18), and that of F9 was the highest (1.42). The results of molybdenum-stibium colorimetry confirmed that eight strains could solubilize Ca_3_(PO_4_)_2_. F9 had the strongest phosphate-solubilizing ability (186.41 mg·L^−1^). Therefore, F9 was selected as a test strain for further research.

### Identification of Phosphate-Solubilizing Fungus

**Figure 2:**
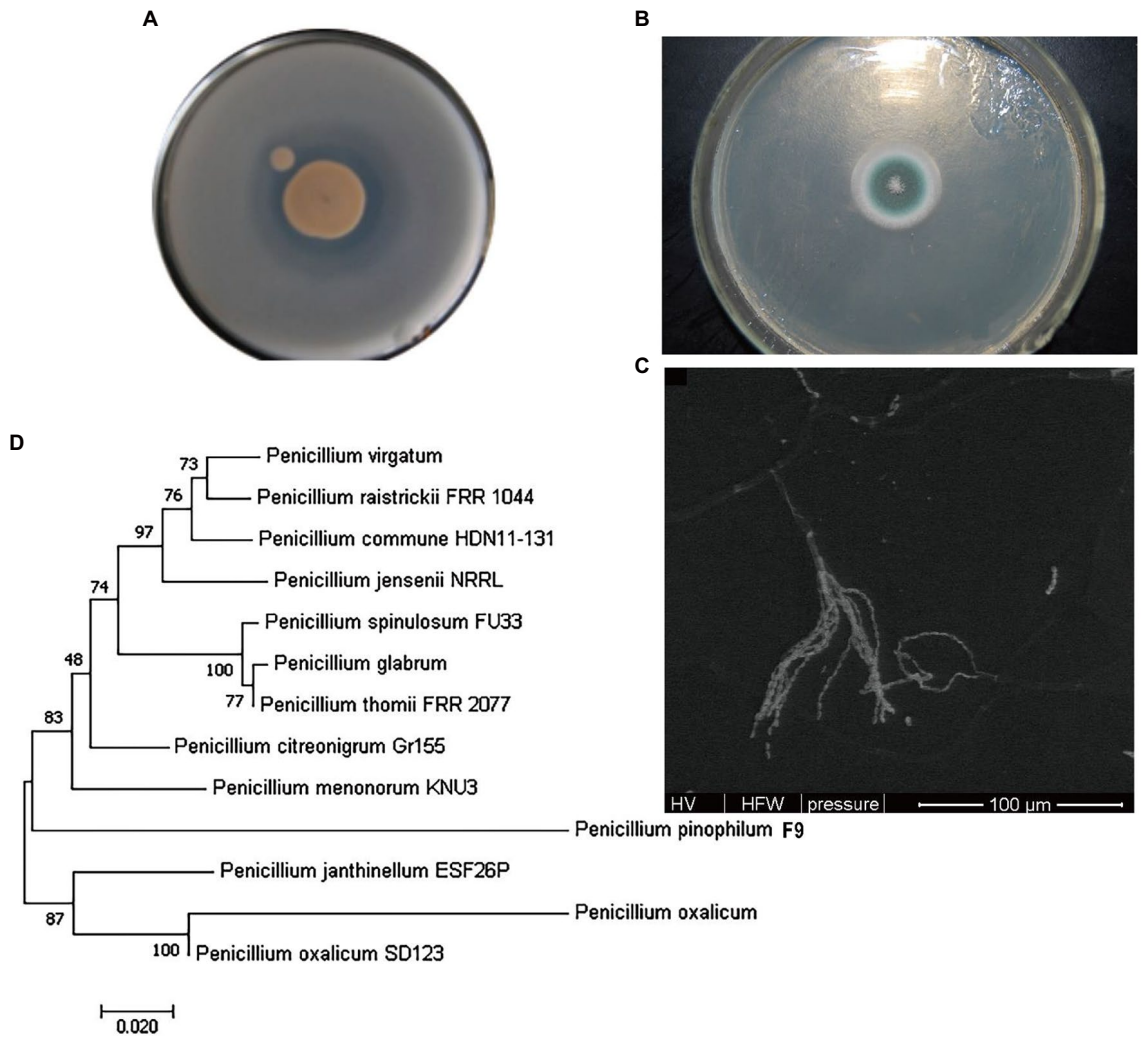
Identification of phosphate-solubilizing fungus F9. **(A)** Colony of F9. **(B)** Solubilized (halo) zone of F9. **(C)** Electron micrograph of F9. **(D)** Phylogenetic tree of F9.

Phosphate-solubilizing fungus F9 was inoculated onto the PDA medium and cultured at 28°C for 120 h, and the colony morphological characteristics are shown in Figure 2A. The F9 colonies on the PDA plate were white at first, gradually turned dark green and finally became grayish green, with white edges, were flat and floss-shaped, and conidia shed off easily. Obvious solubilized (halo) zones emerged on the PSFM plate (Figure 2B). The structure of phosphate-solubilizing fungus F9 observed under the electron microscope is shown in Figure 2C. The conidiophores of F9 were found in the substrate, and the penicillus was composed of verticillated sterigma of conidiophores, showing single verticillation. According to the contrast analysis in line with Fungal Identification Manual, fungus F9 conformed to the growth characteristics of *Penicillium*.

The molecular identification of F9 was performed using the ITS technique. The ITS sequence of F9 was obtained by PCR amplification, and the GenBank ID was KT351451. The results of BLAST analysis revealed that this sequence was 99% homologous to the ITS sequence of related *Penicillium* strains published. The phylogenetic tree based on the *Penicillium* rDNA-ITS sequence was constructed using MEGA7.0 software (Figure 2D). It was found that the evolutionary distance between phosphate-solubilizing fungus F9 and *P. oxalicum* SD123 KP639194.1 was the shortest. Combined with the morphological characteristics and the structural characteristics of conidia, the phosphate-solubilizing fungus F9 was preliminarily determined as *P. oxalicum*.

### Analysis of Organic Acids in the Culture Medium of *Penicillium oxalicum* F9 During Growth

**Figure 3:**
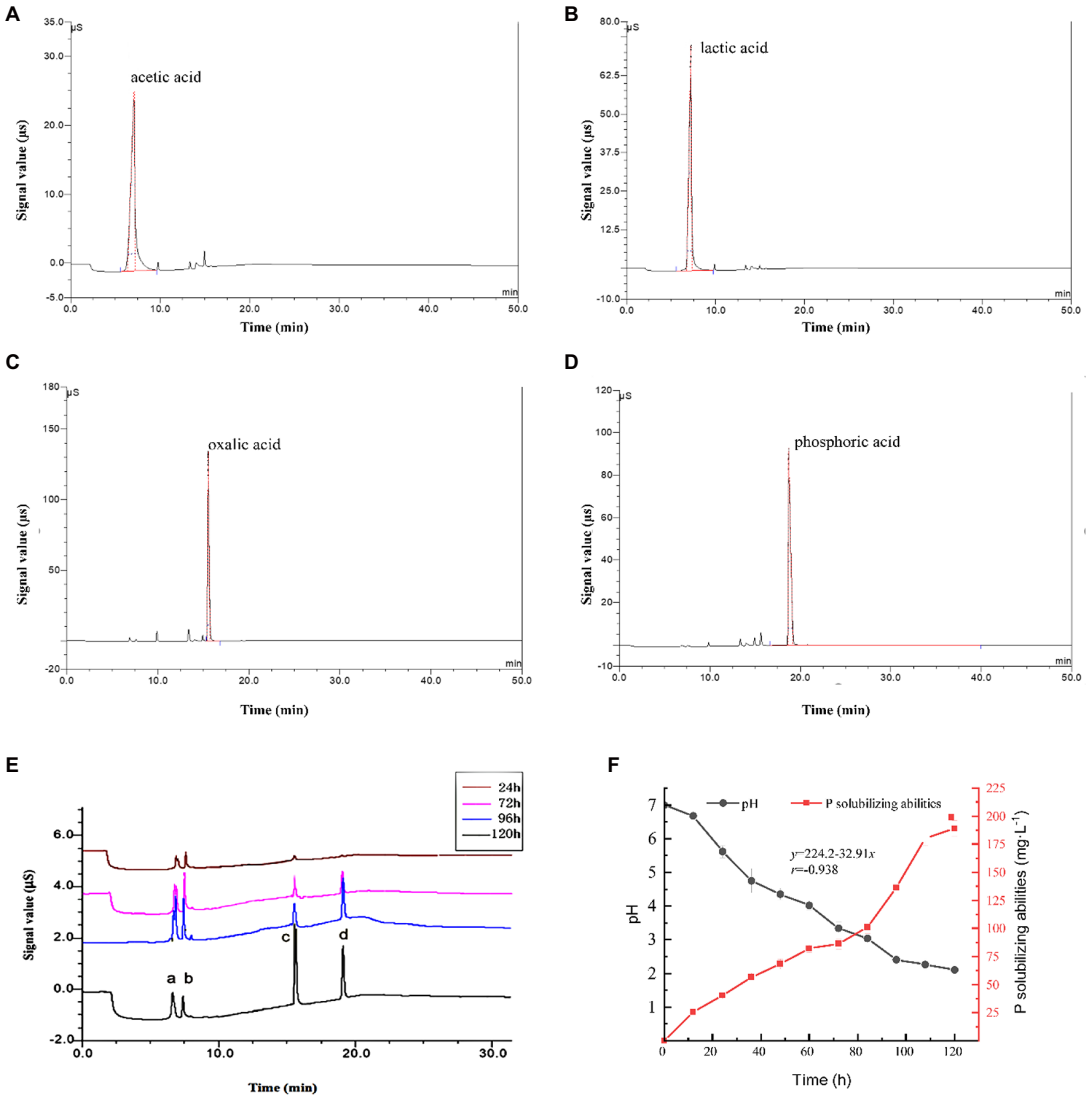
*Penicillium oxalicum* F9 phosphorus-solubilizing ability. **(A–D)**. The ion chromatograms of lactic acid, acetic acid, oxalic acid, and phosphoric acid standard samples, respectively. **(E)** Ion chromatography of *P. oxalicum* F9 culture medium during growth, a, b, c, and d is lactic acid, acetic acid, oxalic acid, and phosphoric acid, respectively. **(F)** Culture medium pH and P-solubilizing capacity of *P. oxalicum* F9.

Ion chromatograms for the culture medium of phosphate-solubilizing *P. oxalicum* F9 cultured in the PSFM for 24, 72, 96, and 120 h are shown in Figure 3E. Obviously, it shows solubilization of calcium phosphate in the culture medium of *P. oxalicum* F9 at various time points during growth. There were four obvious absorption peaks (6.60, 7.30, 15.60, and 19.10 min of elution time) in the culture medium during solubilization of calcium phosphate by *P. oxalicum* F9. Furthermore, it was found that the retention times of the four peaks a, b, c, and d (Figure 3E) were the same as those of the single standard samples of lactic acid, acetic acid, oxalic acid, and phosphoric acid, respectively (Figures 3A–D). The absorption peaks of lactic acid, acetic acid, and oxalic acid were low after 24 h of growth and were gradually increased during additional growth. The absorption peaks of lactic acid and acetic acid were the highest after 96 h, whereas oxalic acid had the highest absorption peak after 120 h of growth. Lactic acid and acetic acid had higher absorption peaks than oxalic acid after 24, 72, and 96 h of growth, whereas oxalic acid had a remarkably higher absorption peak than lactic acid and acetic acid after 120 h of growth. The absorption peak (corresponding to phosphoric acid) was not detected on prophase, but the absorption peak increased gradually with the increase in fermentation time. The reason may be that the total amount of organic acids is low in the early stage and the ability of organic acids to dissolve insoluble calcium phosphate to release soluble phosphorus is limited. With the extension of culture time, the total amount of organic acids secreted by *P. oxalicum* F9 gradually increased, resulting in the increase in phosphate release. The content of organic acids in different stages is shown in Table 1.

**Table 1:** Organic acids in the *Penicillium oxalicum* F9 culture medium.

Duration of growth	Organic acids (mmol·L^−1^)			
	Lactic acid	Acetic acid	Oxalic acid	Total acids
24 h	0.023 ± 0.003d	0.015 ± 0.003c	0.010 ± 0.001c	0.047 ± 0.008c
72 h	0.033 ± 0.001c	0.051 ± 0.004a	0.033 ± 0.005b	0.139 ± 0.013b
96 h	0.054 ± 0.002a	0.053 ± 0.003a	0.047 ± 0.009b	0.153 ± 0.014a
120 h	0.040 ± 0.001b	0.038 ± 0.003b	0.064 ± 0.004a	0.163 ± 0.003a

*The significance letters in each column indicate significant differences in various stages of growth*.

During solubilization of calcium phosphate by *P. oxalicum* F9, the concentration of lactic, acetic, and oxalic acids, as well as total acids showed an increasing trend when incubation time was prolonged. After 24 h, the concentrations of lactic acid and acetic acid were 0.023 mmol·L^−1^ and 0.015 mmol·L^−1^, respectively, whereas the concentration of oxalic acid (0.010 mmol·L^−1^) was significantly lower than that of lactic acid and acetic acid (*p* < 0.05). With the increased culturing time, the concentration of lactic, acetic, and oxalic acids, as well as total acids displayed an upward trend. The concentration of lactic acid (0.054 mmol·L^−1^) and acetic acid (0.054 mmol·L^−1^) was highest after 96 h of culturing, and that of oxalic acid (0.064 mmol·L^−1^) peaked after 120 h of culturing. Furthermore, the concentration of oxalic acid was higher than that of lactic acid (by 0.024 mmol·L^−1^) and acetic acid (by 0.026 mmol·L^−1^).

Meanwhile, the pH of the culture medium gradually declined with an increase in culturing time during solubilization of calcium phosphate by *P. oxalicum* F9 (Figure 3F). At 108 and 120 h, the pH tended to be stable and dropped to 2.40. The phosphate-solubilizing effect was enhanced with the prolonged culturing time. The phosphate-solubilizing ability of *P. oxalicum* F9 on calcium phosphate reached 180–190 mg·L^−1^ after 108 h. During solubilization of calcium phosphate by *P. oxalicum* F9, there was a correlation between the culture medium pH and phosphate-solubilizing ability. The lower the pH of the culture medium was, the stronger the phosphate-solubilizing effect would be, showing a negative correlation (*r* = −0.938).

### Phosphate-Solubilizing Effect of *Penicillium oxalicum* F9 on the Different Phosphorus Sources

To evaluate the phosphate-solubilizing effect of *P. oxalicum* F9 on the different insoluble phosphorus sources, except for, the other insoluble phosphorus sources AlPO_4_ and FePO_4_ were utilized in our research (Table 2). The total acid concentration in medium was 0.143 mmol·L^−1^ for insoluble phosphorus sources Ca_3_(PO_4_)_2_; however, the total acid concentrations for AlPO_4_ and FePO_4_ were 0.073 and 0.089 mmol·L^−1^, lower than the insoluble phosphorus sources Ca_3_(PO_4_)_2_. And the solubilizing ability of *P. oxalicum* F9 on Ca_3_(PO_4_)_2_ was 189.10 mg·L^−1^ and significantly higher than the other two insoluble phosphorus sources AlPO_4_, 40.83 mg·L^−1^, and FePO_4_, 72.11 mg·L^−1^ (*p* < 0.05). This result indicated that the phosphate-solubilizing ability of *P. oxalicum* F9 on Ca_3_(PO_4_)_2_, as a phosphorus source, was greater than on AlPO_4_ and FePO_4_. Consequently, to better investigate the phosphorus solubilization ability of *P. oxalicum* F9 and its mutant strains, the following study used Ca_3_(PO_4_)_2_, as a source of insoluble phosphorus.

**Table 2:** The phosphate-solubilizing ability of *Penicillium oxalicum* F9 in different phosphorus sources.

Phosphorus source	Organic acid (mmol·L^−1^)				P-solubilizing abilities (mg·L^−1^)
	Lactic acid	Acetic acid	Oxalic acid	Total acid	
Ca_3_(PO_4_)_2_	0.040 ± 0.001a	0.038 ± 0.003a	0.064 ± 0.004a	0.143 ± 0.010a	189.10 ± 6.21a
AlPO_4_	0.021 ± 0.002b	0.020 ± 0.004c	0.052 ± 0.005b	0.073 ± 0.010c	40.83 ± 4.30c
FePO_4_	0.010 ± 0.010c	0.037 ± 0.008b	0.042 ± 0.002c	0.089 ± 0.008b	72.11 ± 3.92b

*The significance letters in each column indicate significant differences in different phosphorus sources*.

### Ion-Beam Mutagenesis of *Penicillium oxalicum* F9

**Figure 4:**
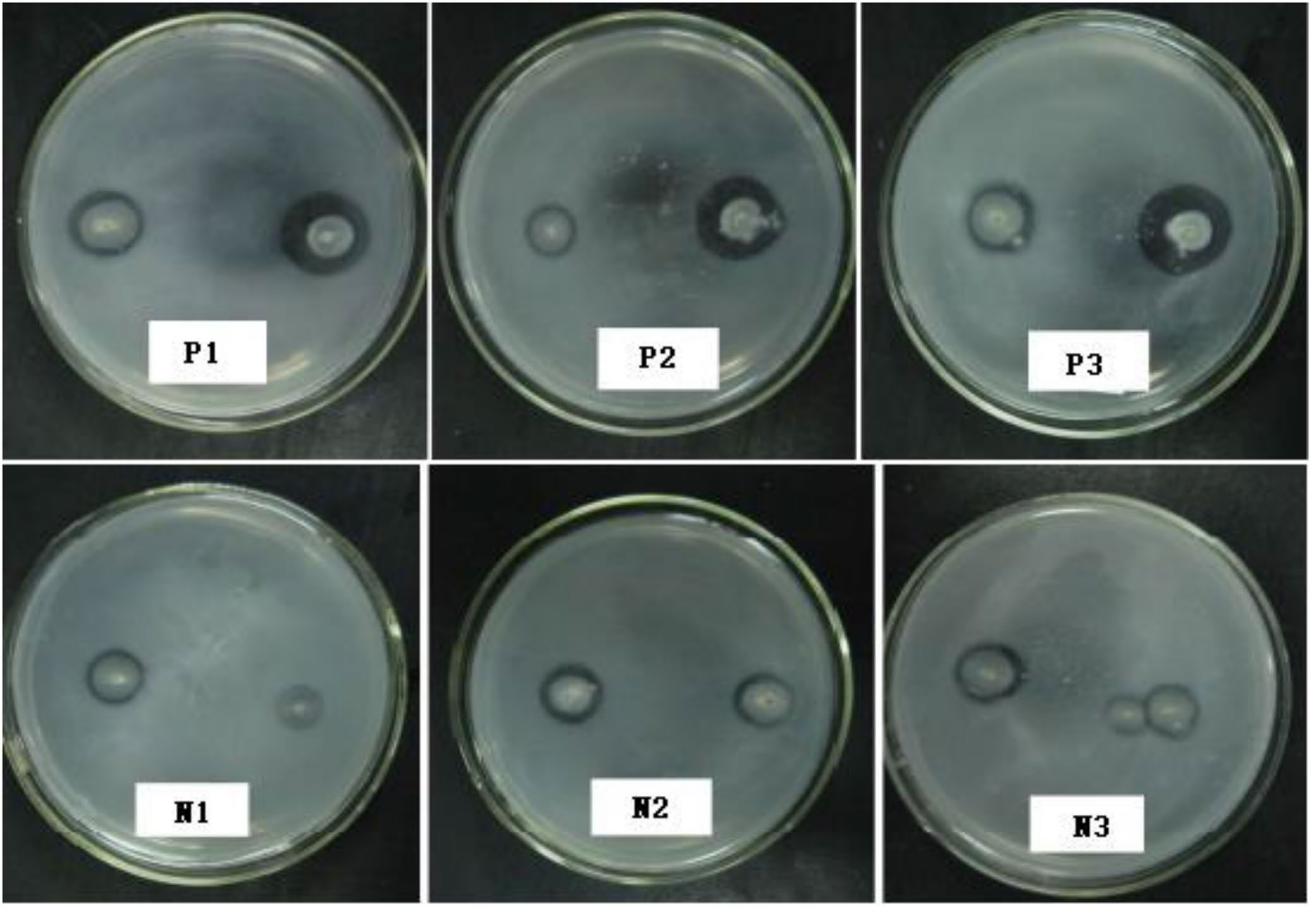
Soluble (halo) zones of mutants after the second round of mutagenesis.

The *P. oxalicum* F9 spores were subjected to the first round of mutagenesis *via* low-energy nitrogen ion radiation at the doses of 30 keV, 1 × 10^15^ ions·cm^−2^ and 25 keV, 1 × 10^16^ ions·cm^−2^. The fungal strains were initially screened by assessing the solubilized (halo) zones (Figure 4) on the solid PSFM plates and rescreened *via* culturing in the liquid medium on a shaker for 120 h; as a result, we identified six phosphate-solubilizing *Penicillium* strains with more than 20% increased phosphate-solubilizing ability and nine strains with 20% decreased phosphate-solubilizing ability (Table 3). The phosphate-solubilizing ability of positive mutant strain p-1 was enhanced most significantly (235.70 mg·L^−1^), up by 26.40% compared with the control strain. The phosphate-solubilizing ability of negative mutant strain n-1 declined most significantly (133.80 mg·L^−1^), down by 28.20% compared with the control strain. The p-1 and n-1 strains were subjected to the second round of mutagenesis under the same radiation conditions. Six mutant strains varying in phosphate-solubilizing ability by more than 30% were obtained, including three positive (P1, P2, and P3) and three negative (N1, N2, and N3) mutant strains. The soluble (halo) zones of the six mutant strains are shown in Figure 4 and Table 3 displays the quantification of the phosphate-solubilizing ability. The soluble (halo) zones of positive mutant strains (P1, P2, and P3) of *Penicillium* strains were greater compared with those of the control strains, whereas the negative mutant strains (N1, N2, and N3) had substantially smaller soluble (halo) zones compared with the control strains (Figure 4). Compared with that of control strains, the phosphate-solubilizing ability of P1, P2, and P3 was enhanced by 56.88%, 42.26%, and 32.15%, and that of N1, N2, and N3 was weakened by 47.53%, 35.27%, and 30.86%, respectively.

**Table 3:** Positive and negative mutants of *Penicillium oxalicum* F9 obtained by low-energy ion beam mutagenesis.

Mutagenesis	Mutants	*D*_solvent zones_/*D*_colonies_	P-solubilizing capacity (mg·L^−1^)	Change in P-solubilizing capacity (%)
	F9	1.24 ± 0.05	186.41 ± 3.20	0
	p-1	1.66 ± 0.06	235.70 ± 3.59	+26.41 ± 1.93
	p-2	1.49 ± 0.06	228.20 ± 2.61	+22.41 ± 1.41
	p-3	1.45 ± 0.02	225.92 ± 4.88	+21.23 ± 2.62
	p-4	1.49 ± 0.04	225.01 ± 4.27	+20.71 ± 2.29
	p-5	1.48 ± 0.05	224.13 ± 4.43	+20.20 ± 2.38
	p-6	1.47 ± 0.08	223.91 ± 6.25	+20.12 ± 3.35
First round	n-1	1.04 ± 0.04	133.80 ± 3.13	−28.21 ± 1.68
	n-2	1.08 ± 0.03	141.92 ± 2.66	−23.92 ± 1.43
	n-3	1.12 ± 0.07	145.51 ± 4.60	−22.03 ± 2.47
	n-4	1.14 ± 0.05	147.31 ± 4.41	−21.01 ± 2.37
	n-5	1.12 ± 0.06	147.72 ± 2.88	−20.80 ± 1.54
	n-6	1.10 ± 0.02	148.40 ± 3.54	−20.41 ± 1.90
	n-7	1.12 ± 0.06	148.92 ± 3.15	−20.11 ± 1.69
	n-8	1.11 ± 0.07	149.21 ± 3.19	−20.03 ± 1.71
	n-9	1.10 ± 0.01	149.11 ± 1.39	−20.02 ± 0.75
	P1	2.02 ± 0.06	291.81 ± 5.50	+56.88 ± 2.96
	P2	1.92 ± 0.06	264.62 ± 10.21	+42.26 ± 5.48
Second round	P3	2.12 ± 0.04	245.80 ± 8.72	+32.15 ± 4.67
	N1	1.00 ± 0.04	97.61 ± 4.41	−47.53 ± 2.36
	N2	1.03 ± 0.02	120.40 ± 7.21	−35.27 ± 3.87
	N3	1.02 ± 0.03	128.60 ± 8.30	−30.86 ± 4.46

Analysis of organic acids and pH of culture medium of mutant strains of phosphate-solubilizing *P. oxalicum* F9.

**Figure 5:**
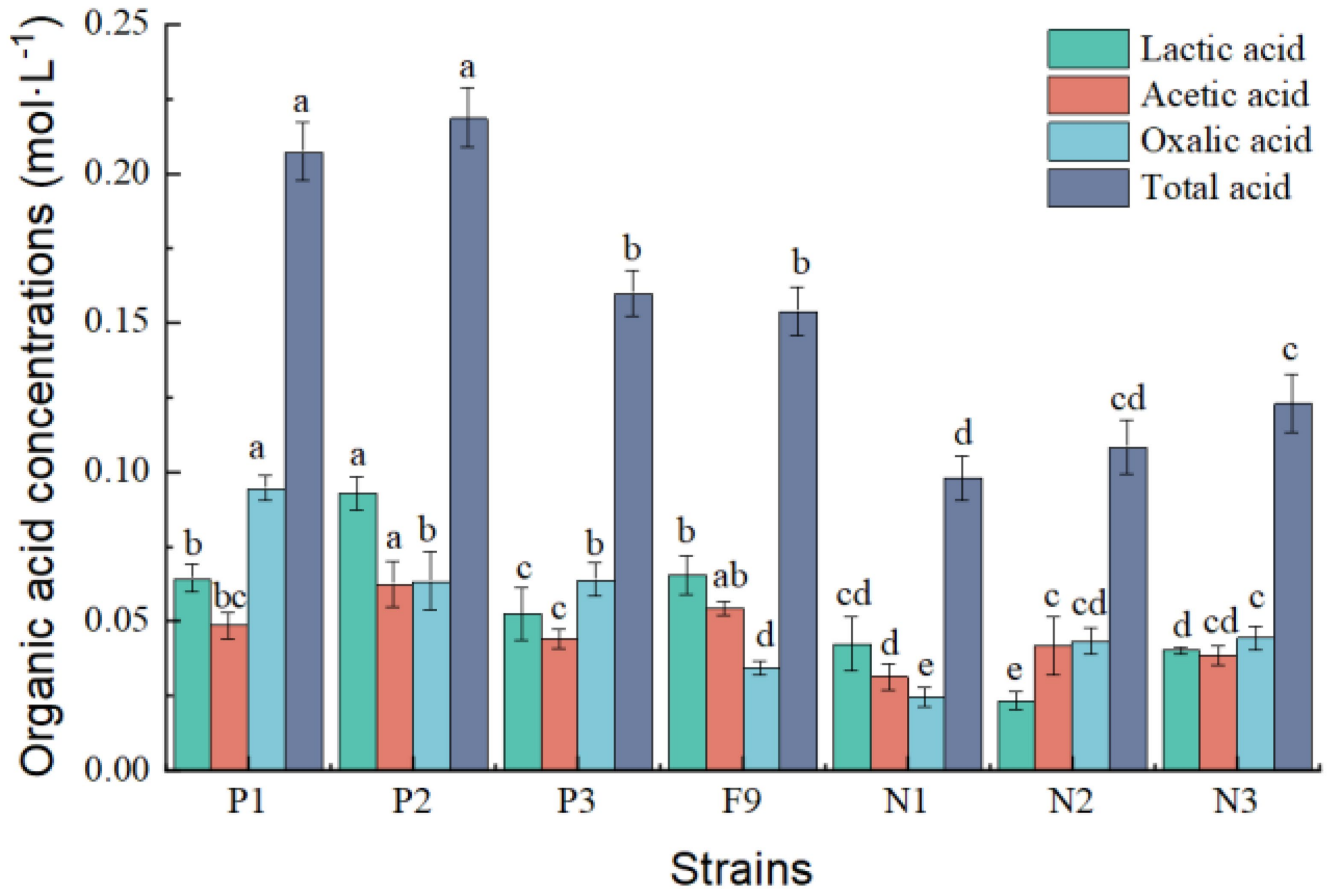
Organic acid content in PSFM medium of *Penicillium oxalicum* F9 and mutants. a, b, c, d, and e indicate significant differences in various strains.

**Figure 6:**
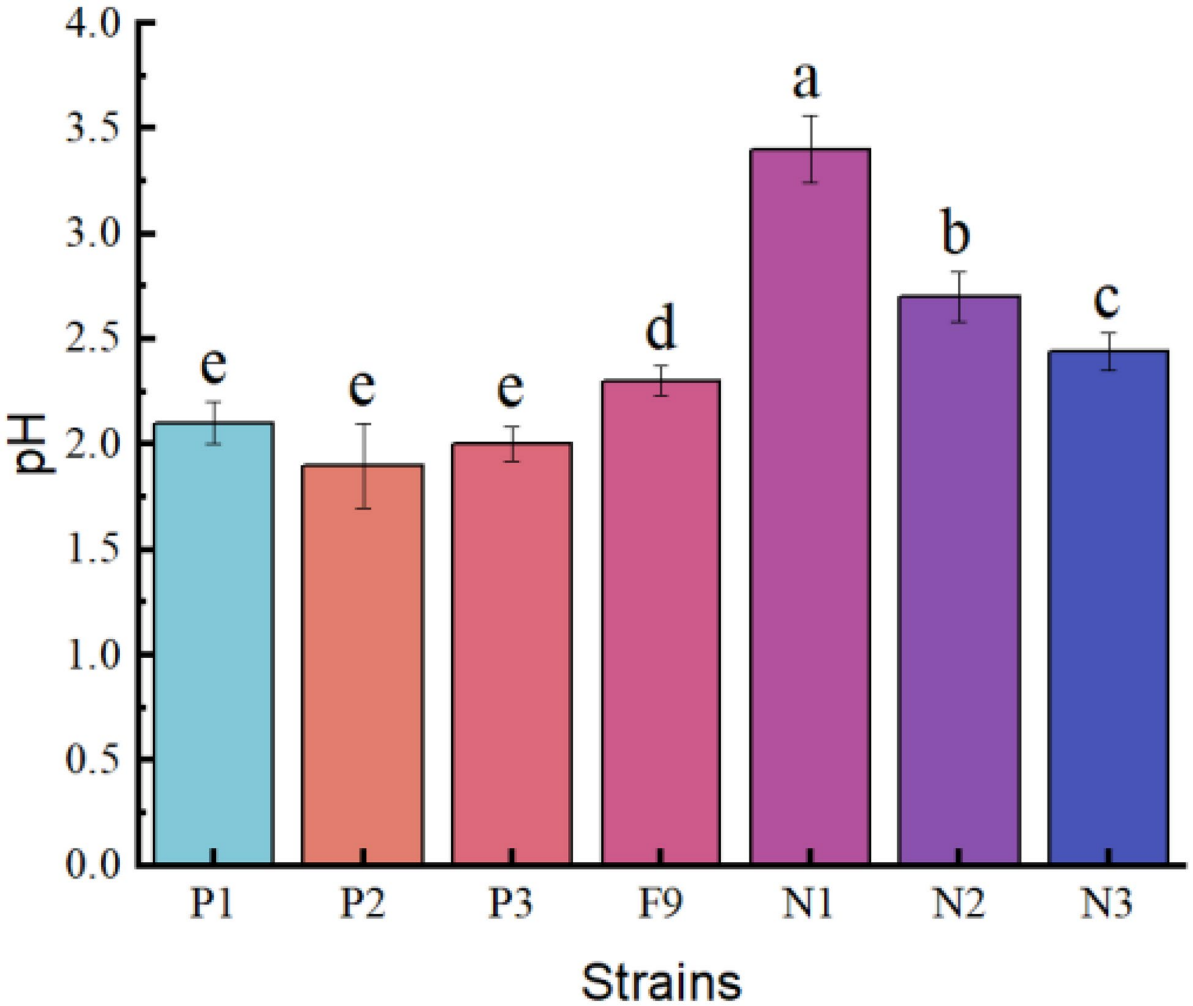
The pH of the PSFM culture medium of *Penicillium oxalicum* F9 and mutants. a, b, c, d, and e indicate significant differences in various strains.

After the *P. oxalicum* F9 strain and the mutant strains (P1, P2, and P3 as well as N1, N2, and N3) were cultured in the PSFM for 120 h, the organic acid concentrations and pH of the culture medium were analyzed (Figures 5, 6).

The total amounts of organic acids secreted by the three positive mutant strains P1, P2, and P3 were 0.207, 0.218, and 0.160 mmol·L^−1^, respectively, representing increases of 68.73%, 77.85%, and 30.13%, respectively, compared with the original strain *P. oxalicum* F9 (*p* < 0.05). The total amounts of organic acids secreted by the three negative mutant strains N1, N2, and N3 were 0.098, 0.108, and 0.114 mmol·L^−1^, respectively, representing decreases of 20.36%, 11.89%, and 7.41%, respectively, compared with the original *P. oxalicum* F9 strain (*p* < 0.05; Figure 5). The phosphate-solubilizing ability of positive mutant strains P1, P2, and P3 was enhanced by 56.88%, 42.26%, and 32.15%, whereas that of negative mutant strains N1, N2, and N3 was weakened by 47.53%, 35.27%, and 30.86%, respectively, compared with *P. oxalicum* F9 (Table 2). The larger the total amount of organic acids secreted by the strains, the stronger their phosphate-solubilizing ability would be.

The pH of the culture medium of the positive mutant strains P1, P2, and P3 was 2.10, 1.91, and 2.02, respectively, and there was no significant difference among the three (*p* > 0.05). However, the pH of the culture medium of the three mutant strains was significantly lower than that of the *P. oxalicum* F9 (*p* < 0.05). The capacity of the three mutant strains to acidify the growth medium was stronger than that of the *P. oxalicum* F9. The pH of the culture medium of inoculation strains N1, N2, and N3 was 3.42, 2.71, and 2.44, respectively. The pH of the culture medium of the three mutant strains was significantly higher than that of the original *P. oxalicum* F9 strain (*p* < 0.05). The capacity of the three mutant strains to acidify the growth medium was weaker than that of the *P. oxalicum* F9. The three positive mutant strains had lower pH, and the three negative mutant strains had higher pH than *P. oxalicum* F9. Based on the phosphate-solubilizing ability of *P. oxalicum* F9 and the mutant strains (Table 2), it can be inferred that the phosphate-solubilizing ability of phosphate-solubilizing *P. oxalicum* F9 had a negative correlation with the pH of the culture medium.

## Discussion

Currently, there have been about 60 types of phosphate-solubilizing fungi identified (Busato et al., 2017), and they mainly belong to *Alternaria*, *Aspergillus*, *Fusarium*, *Penicillium*, *Talaromyces*, and *Trichoderma* (Doilom et al., 2020). *Aspergillus* and *Penicillium* have widely reported fungi with a strong phosphate-solubilizing ability (Kucey, 1983; Hao et al., 2021). Lin et al. (2001) conducted a comparative study on the phosphate-solubilizing ability of some bacteria and fungi and found that the capacity of bacteria to dissolve phosphate rock powder ranged from 26.92 to 43.34 mg·L^−1^, whereas the capacity of most fungi was 59.64–145.36 mg·L^−1^; hence, fungi have a stronger capacity to dissolve phosphate rock powder than bacteria. Nath et al. (2012) found two strains of *Penicillium* with a strong phosphate-solubilizing ability (39.22 and 86.10 mg·L^−1^). In the current study, nine strains of phosphate-solubilizing fungi were isolated from the rhizospheric soil of major crops wheat and cotton in the downstream region of the Yellow River. Among them, *P. oxalicum* F9 had the strongest phosphate-solubilizing ability, and it was identified by morphological and molecular characterization that the strain was *Penicillium oxalicum* with the high phosphate-solubilizing activity (186.41 mg·L^−1^), higher than that of bacteria and fungi of the same type reported previously. The *P. oxalicum* F9 isolated in this study provides important material for the research on the phosphate-solubilizing mechanism and application of phosphate-solubilizing fungi.

Low-energy ion beam mutagenesis leads to single base substitution and DNA double-strand breakage (Thopan et al., 2017), and a large number of free radicals will be produced. Mutation breeding of highly efficient phosphate-solubilizing fungi microorganisms and characterization of the underlying phosphate-solubilizing mechanism has always been hot research topics in the field of soil microorganisms and biofertilizers (Guo et al., 2019). The phosphate-solubilizing effect of microorganisms can be enhanced by radiation mutagenesis. Silva et al. (2014) induced mutation of *Aspergillus niger via* UV and confirmed that the phosphate-solubilizing ability of mutant FS1-331 was increased by 1.70 times compared with that of wild strains. In recent years, low-energy ion beam mutagenesis for microorganisms has been extensively researched (You et al., 2009; Li et al., 2011a; Zhang et al., 2016; Semsang et al., 2018). Semsang et al. (2018) used low-energy N ion beam mutagenesis to acquire a mutant which had improved yields and seed quality. In this study, *P. oxalicum* F9 was subjected to two rounds of low-energy nitrogen ion mutagenesis. The three positive mutants (P1, P2, and P3) obtained by ion beam mutagenesis had significantly higher phosphorus solubilization abilities than the control strain (56.88%, 42.26%, and 32.15%); in contrast, the three negative mutants exhibited significantly lower phosphorus solubilization abilities than the control strain. In addition, the acidification and organic acid secretion by the positive mutant strains obtained by ion beam mutagenesis were markedly stronger than those of *P. oxalicum* F9, but they were weaker in the negative mutant strains than those of *P. oxalicum* F9 (Figures 5, 6). Ion beam mutagenesis can alter the acidification, organic acid-secreting, and phosphate-solubilizing capacities of *P. oxalicum* F9. In summary, numerous phosphate-solubilizing mutants were induced *via* low-energy ion implantation, which provided valuable test materials for breeding of highly efficient phosphate-solubilizing microorganisms and exploration of the underlying phosphate-solubilizing mechanism in *P. oxalicum* F9.

Secreting organic acids is a common phenomenon and also the main reason for the phosphate-solubilizing effect by phosphate-solubilizing microorganisms that can secrete organic acids to lower the growth medium pH, thereby solubilizing insoluble inorganic phosphorus (Alori et al., 2017; Tomer et al., 2017; Zhang et al., 2018). Most studies showed a significant correlation between the phosphate-solubilizing ability of phosphate-solubilizing bacteria and the pH of fermentation liquid. In this study, the capacity of *P. oxalicum* F9 to solubilize calcium phosphate in the PSFM was significantly and negatively correlated with the pH of the culture medium (*r* = −0.938). There was also a similar relation between the phosphate-solubilizing ability of mutant strains obtained by ion beam mutagenesis and the pH. The pH of the culture medium of the positive mutant strains (P1, P2, and P3) at 120 h (pH 2.40) was significantly lower than that of *P. oxalicum* F9 (*p* < 0.05), whereas the pH of the culture medium of the negative mutant strains (N1, N2, and N3) at 120 h was evidently higher than that of *P. oxalicum* F9. The phosphate-solubilizing ability of the mutant strains of phosphate-solubilizing *P. oxalicum* F9 was negatively correlated with the pH of their culture medium.

The organic acids secreted by phosphate-solubilizing *P. oxalicum* F9 form complexes or chelates with calcium ions of water-insoluble calcium phosphate so that the phosphate ions are released. During solubilization of calcium phosphate in the PSFM by *P. oxalicum* F9 and its mutants, the three types of organic acids (lactic acid, acetic acid, and oxalic acid) were secreted. It is speculated that these three types are the main organic acids involved in phosphate solubilization by *P. oxalicum* F9. During the growth of *P. oxalicum* F9 and solubilization of calcium phosphate, the total amount of organic acids secreted increased, and the phosphate-solubilizing effect also became more obvious with the increased duration of growth. In this study, the concentration of lactic acid and acetic acid in the culture medium was significantly higher than that of oxalic acid after 24 h of growth (*p* < 0.05), whereas the concentration of oxalic acid was significantly higher than that of lactic acid and acetic acid after 120 h of growth (*p* < 0.05). It is speculated that the phosphate-solubilizing effect of *P. oxalicum* F9 was dominated by lactic acid and acetic acid after 24 h of growth, but it is dominated by oxalic acid after 120 h of growth. The close relation between the total amount of organic acids secreted by *P. oxalicum* F9 and its phosphate-solubilizing effect was also confirmed in the mutant strains. The total amount of organic acids of the three positive mutant strains (P1, P2, and P3) was significantly larger than that of the *P. oxalicum* F9, whereas the three negative mutant strains (N1, N2, and N3) secreted significantly less organic acids than *P. oxalicum* F9. Consequently, a stronger capacity of *P. oxalicum* F9 to secrete organic acids will result in a greater capacity of it to solubilize phosphate.

## Conclusion

In this study, a phosphorus-solubilizing fungus *P. oxalicum* F9 was isolated from the rhizospheric soil under wheat and cotton fields in the lower reaches of the Yellow River. *P. oxalicum* F9 dissolved water-insoluble calcium phosphate by secreting lactic acid, acetic acid, and oxalic acid. And the secretion of the above three organic acids gradually increased with the extension of the culture time with pH values of the medium decreased correspondingly. In addition, the organic acids formed complexes or chelates with calcium ions, so that the phosphate was released. Furthermore, compared with the original strain, the high-efficiency phosphorus-solubilizing mutant obtained by ion beam mutagenesis secreted more organic acids and had a stronger phosphorus-solubilizing effect. Overall, this study provides a theoretical basis and a new direction for the study and development of the phosphorus-solubilizing mechanism of phosphorus-solubilizing fungi.

## Data Availability Statement

The raw data supporting the conclusions of this article will be made available by the authors, without undue reservation.

## Author Contributions

TY, LL, BW, JT, SZ, and ZW designed and performed research study. TY, LL, SZ, and FS wrote the paper. All authors contributed to the article and approved the submitted version.

## Funding

This research was supported by National Natural Science Foundation of China (11305047) and Science and Technology Program of Henan Province (222102110122).

## Conflict of Interest

The authors declare that the research was conducted in the absence of any commercial or financial relationships that could be construed as a potential conflict of interest.

## Publisher’s Note

All claims expressed in this article are solely those of the authors and do not necessarily represent those of their affiliated organizations, or those of the publisher, the editors and the reviewers. Any product that may be evaluated in this article, or claim that may be made by its manufacturer, is not guaranteed or endorsed by the publisher.
